# Tracing the Impact of Public Health Interventions on HIV-1 Transmission in Portugal Using Molecular Epidemiology

**DOI:** 10.1093/infdis/jiz085

**Published:** 2019-02-26

**Authors:** Tetyana I Vasylyeva, Louis du Plessis, Andrea C Pineda-Peña, Denise Kühnert, Philippe Lemey, Anne-Mieke Vandamme, Perpétua Gomes, Ricardo J Camacho, Oliver G Pybus, Ana B Abecasis, Nuno R Faria

**Affiliations:** 1 Department of Zoology, University of Oxford, United Kingdom; 2 New College, University of Oxford, United Kingdom; 3 Center for Global Health and Tropical Medicine, Instituto de Higiene e Medicina Tropical, Universidade Nova de Lisboa; 4 Laboratory of Molecular Biology, LMCBM, SPC, Hospital de Egas Moniz–Centro Hospitalar de Lisboa Ocidental, Lisbon; 5 Center for Interdisciplinary Research Egas Moniz, CiiEM, Almada, Portugal; 6 Molecular Biology and Immunology Department, Fundación Instituto de Inmunología de Colombia; 7 Basic Sciences Department, Universidad del Rosario, Bogotá, Colombia; 8 Max Planck Institute for the Science of Human History, Jena, Germany; 9 Laboratory for Clinical and Epidemiological Virology, Department of Microbiology and Immunology, Rega Institute for Medical Research, KU Leuven, Belgium

**Keywords:** HIV, Portugal, phylodynamics, epidemiology, reproductive number, transmission groups, harm reduction

## Abstract

**Background:**

Estimation of temporal changes in human immunodeficiency virus (HIV) transmission patterns can help to elucidate the impact of preventive strategies and public health policies.

**Methods:**

Portuguese HIV-1 subtype B and G *pol* genetic sequences were appended to global reference data sets to identify country-specific transmission clades. Bayesian birth-death models were used to estimate subtype-specific effective reproductive numbers (R_e_). Discrete trait analysis (DTA) was used to quantify mixing among transmission groups.

**Results:**

We identified 5 subtype B Portuguese clades (26–79 sequences) and a large monophyletic subtype G Portuguese clade (236 sequences). We estimated that major shifts in HIV-1 transmission occurred around 1999 (95% Bayesian credible interval [BCI], 1998–2000) and 2000 (95% BCI, 1998–2001) for subtypes B and G, respectively. For subtype B, R_e_ dropped from 1.91 (95% BCI, 1.73–2.09) to 0.62 (95% BCI,.52–.72). For subtype G, R_e_ decreased from 1.49 (95% BCI, 1.39–1.59) to 0.72 (95% BCI, .63–.8). The DTA suggests that people who inject drugs (PWID) and heterosexuals were the source of most (>80%) virus lineage transitions for subtypes G and B, respectively.

**Conclusions:**

The estimated declines in R_e_ coincide with the introduction of highly active antiretroviral therapy and the scale-up of harm reduction for PWID. Inferred transmission events across transmission groups emphasize the importance of prevention efforts for bridging populations.

## Teaser

Birth-death phylodynamic models uncover how human immunodeficiency virus (HIV) transmission dynamics was decelerated by harm-reduction programs in the 1990s in Portugal. After 2000, transmissions in heterosexuals and people who inject drugs were driving the Portuguese HIV-1 subtype B and G epidemic, respectively.

Human immunodeficiency virus type 1 (HIV-1) subtype B dominates the HIV epidemic in Western European countries [1]. Since the 1980s, the Portuguese HIV epidemic was concentrated in heterosexual (HET) populations and subtype B was the most common strain [2]. However, by 2001 nearly two thirds of reported AIDS cases in Portugal were linked to people who inject drugs (PWID), and the country had the highest HIV incidence among PWID in the European Union [3, 4]. Among patients with newly diagnosed disease in Portugal, 35% are PWID, but this proportion has ranged from 45% before 2004 to 15% after [5]. Although the HIV epidemic among Portuguese PWID was initially associated with subtype B transmission, the proportion of subtype G and CRF14_BG infections increased rapidly in this group [6]. Currently, the HIV molecular profile in Portugal is very diverse, with a variety of HIV-1 subtypes (A, C, D, H, J, and F) and recombinant forms, as well as a higher proportion of HIV-2 infections than in other European settings [1, 2, 6–9].

Besides the introduction of antiretroviral therapy (ART) in 1996, several public health approaches have been used to reduce the HIV incidence in Portugal. Since the early 1990s, Portugal has been at the forefront of the implementation of harm-reduction strategies [10]. Methadone opioid substitution treatment (OST) has been available to PWID since 1977, while buprenorphine OST became available in 1999 [11]. In 2004, OST was extended to pharmacy-based provision, and the first syringe-exchange program was established in 1993. In 2001, Portugal effectively decriminalized drug possession (possession of a small amount of drugs for self-consumption was made an administrative offense, rather than a criminal offense), which reportedly resulted in a reduction in the number of drug-related deaths and possibly a drop in the HIV incidence [5, 12].

The effective reproductive number (R_e_) is defined as the average number of secondary infections caused by an infected individual at a particular time point during an epidemic, when susceptibility in the population has decreased (eg, because of increased immunity in the population or intervention measures). It is often used to describe transmission dynamics over the course of an epidemic. Recent developments in phylodynamic modeling enable genetic and epidemiological modeling to be combined, to quantify R_e_ in HIV-infected populations [13–15]. Yet, R_e_ remains to be estimated for the main subtypes and transmission groups associated with the Portuguese HIV epidemic.

Here, we collate 680 HIV-1 subtype B and G *pol* genetic sequences with known transmission group information from the Portuguese HIV drug resistance database. We use several phylodynamic approaches to investigate temporal changes in R_e_ of the predominant subtypes and circulating clades, and we describe the viral lineages mixing among different transmission groups. Our findings highlight the effect of preventive interventions on the HIV-1 dynamics in Portugal.

## METHODS

### Genetic Data

HIV-1 subtype B and G *pol* genetic sequences were obtained from the Portuguese HIV database. For the purpose of this analysis we used all sequences collected between 2001 and 2013 with available information regarding transmission risk group (self-reported by patients). Reference HIV-1 subtype B sequences were obtained from the SPREAD database (SPREADdb), created by the European Society for Translational Antiviral Research (available at: http://www.esar-society.eu/). The SPREADdb contains annotated sequences generated from patients with newly diagnosed infection during 2001–2013 from 26 European countries [16]. For each Portuguese subtype B sequence, we extracted the 10 most similar sequences from the SPREADdb. For HIV-1 subtype G, we used all publicly available subtype G sequences from the Los Alamos National Laboratory HIV database (LANLdb) [17] as reference sequences (for accession numbers see Supplementary Table 1). We first codon-aligned all sequences, using Clustal Omega [18], and subsequently edited the alignment manually in MEGA7 [19].

### Initial Phylogenetic Analyses

We used the combined subtype B and G data sets (from the Portuguese HIV database, SPREADdb, and LANLdb) to generate maximum likelihood phylogenies for each subtype [20]. We used the Hasegawa-Kishino-Yano nucleotide substitution model with gamma-distributed rate variation among sites (HKY+G). For subtype B, we used Cluster Picker [21] to identify circulating HIV-1 clades; these were defined as the deepest Portuguese-only clades with >10 sequences that exhibited a within-clade genetic distance <6% and a Shimodaira-Hasegawa (SH)–like statistical support >90%. Given that most Portuguese subtype G sequences belonged to a single well-supported clade (SH-like support, 90%), we analyzed all 236 sequences as a single phylogenetic clade. For each clade, we estimated a maximum likelihood tree, using RAxML [22], and quantified temporal signal by regressing the root-to-tip genetic distance of each sequence in the tree with its sampling date, as implemented in Tempest [23].

### **Estimating** R_e_**for HIV Lineages**

We used birth-death models implemented in BEASTv2.4 [24] to estimate time-varying rates of epidemic spread, measured as changes in R_e_, denoted R_e_(*t*). In this model, lineages are added to the tree upon infection, at rate λ (the birth rate), and are removed, at rate δ (the death rate), upon becoming noninfectious (either through treatment, isolation, or death). We assumed that individuals became noninfectious upon sampling and were removed from the pool of transmitters, since, upon sampling, they received treatment, and their viral load could be reduced to undetectable levels. However, it is important to note that not everyone who receives a diagnosis will achieve virological suppression, and, therefore R_e_, which is here defined by λ/δ (where 1/δ is the average infectious period) represents a lower bound on the true value of R_e_. We also estimated the sampling proportion and the “origin date” of the epidemic (which was older than the time to most recent common ancestor (TMRCA) of the tree reconstructed from the sampled sequences) [25]. In addition, R_e_, δ, and the sampling proportion were allowed to instantaneously change at certain time points (thus these parameters were piecewise constant functions of time).

We used 3 different variations of the birth-death model in our analyses. In model 1 (the constant rate birth-death model), R_e_ was assumed to be constant through time, resulting in an average R_e_ over the course of the epidemic [13]. Model 2 (the birth-death skyline [BDSKY] model) assumed a piecewise constant R_e_ over 10 equidistant intervals, between the TMRCA and the most recent sample [26]. Model 3 (the constant-shift-constant model) assumed a piecewise constant R_e,_ but with only 2 epochs, analogous to a previously described coalescent model [27]. In addition to estimating the average R_e_ during an early and a late epoch, model 3 is able to estimate directly from sequence data the timing of the transition (shift) in epidemic growth rate between the 2 epochs, including for hierarchical model scenarios with multiple trees. We used the TreeSlicer package in BEAST2 for these analyses (available at: https://github.com/laduplessis/skylinetools).

R_e_ estimates were calculated separately for the 2 subtypes. For the subtype B clades, we used a hierarchical phylogenetic approach across all subtype B clades, in which the molecular clock, nucleotide substitution, and birth-death tree prior model parameters were all linked across clades [28, 29]. We also analyzed all 5 HIV-1 subtype B clades as a single phylogenetic tree (Supplementary Materials). For all BEAST analyses, mixing of the Markov chain Monte Carlo (MCMC) chains was visually inspected in Tracer [30]. Analysis were typically run for 100–200 million MCMC steps, and effective sample sizes were >200 for all parameters. We used the bdskytools package in R (available at: https://github.com/laduplessis/bdskytools) to plot the results of BDSKY analyses (model 2).

### Discrete Trait and Association Index (AI) Analyses

To characterize population structure according to transmission groups, we estimated the phylogenetically based AI statistic that quantifies the association between a phylogeny and trait values associated with each taxon. We quantified AI, which ranges from 0 to 1 (0 represents maximum population structure, and 1 indicates panmixia), using 3 independent approaches implemented in BEAST, BaTS, and HyPhy [31–33] (Supplementary Materials).

To describe and quantify viral movement between different transmission risk groups, we performed a discrete trait diffusion analysis (DTA), using transmission group as a discrete trait [34], for clades with significant transmission group structure. Initially, each sequence was annotated with one of the 5 known transmission groups, as follows: men who sex with men (MSM), PWID, HET, mother-to-child transmission (MTC), or Other (eg, blood transfusion). To count the expected number of virus lineage movements among transmission groups, we used a robust counting (Markov jumps) approach [35, 36]. Based on preliminary analyses, we next considered 3 transmission groups (with the 2 transmission groups that contributed the highest proportion of transmission group transition events in a given data set and with all other transmission groups included in a new category called “Other”). For both analyses, we used a Bayesian stochastic search variable selection procedure to estimate the most relevant transition pathways among transmission groups. Statistical support was measured using Bayes factors (BF) [37] and summarized using SpreaD3 [38]. We assumed that a BF > 10 is strong evidence for a well-supported viral pathway between 2 transmission groups [37].

Additional methodological details can be found in the Supplementary Materials. All xml files are accessible online at the GitHub repository (available at: https://github.com/HIVMolEpi/HIV-1-Portugal).

## RESULTS

### Genetic Sequences

A total of 3462 HIV-1 subtype B and 2678 subtype G *pol* genetic sequences were available from the Portuguese HIV database. Of these, self-reported transmission group information was available for 444 subtype B and 236 subtype G sequences. The transmission risk group distribution of sequences in this analysis is very similar to that reported in the Portuguese HIV/AIDS annual report, apart from the proportion of MTC sequences (13% in our analysis vs 1% in the report; Supplementary Table 2) [5].

**Figure 1.**
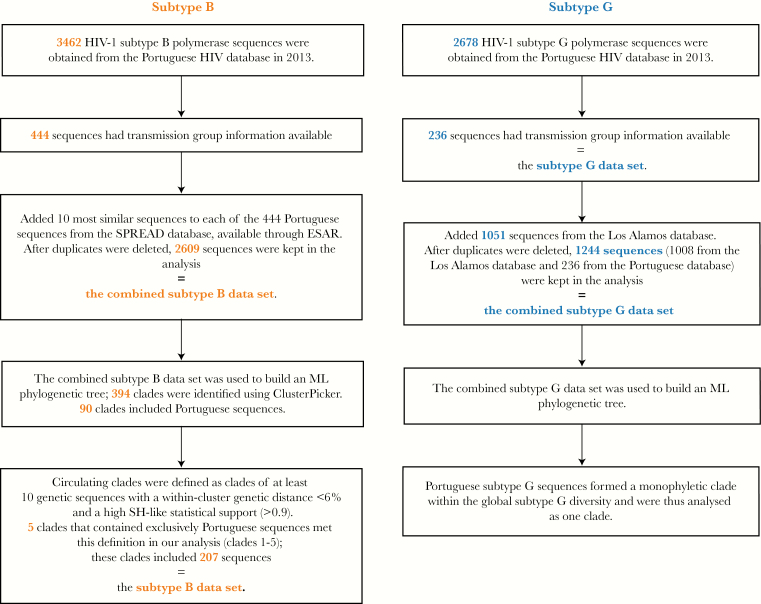
Diagram describing the process of selecting human immunodeficiency virus type (HIV-1) subtype B and G sequences for the analysis. ML, maximum likelihood; SH, Shimodaira-Hasegawa.

Additionally, we analyzed 2165 unique reference subtype B sequences from the SPREADdb and 1008 unique subtype G reference sequences from the LANLdb. Data sets that include both Portuguese and reference sequences are hereafter referred to as “combined” data sets. The combined data sets therefore comprised 2609 subtype B sequences (2165 from the SPREADdb and 444 from the Portuguese database) and 1244 subtype G sequences (1008 from the LANLdb and 236 from the Portuguese database; Figure 1).

### Phylogenetic Clade Analysis

**Figure 2.**
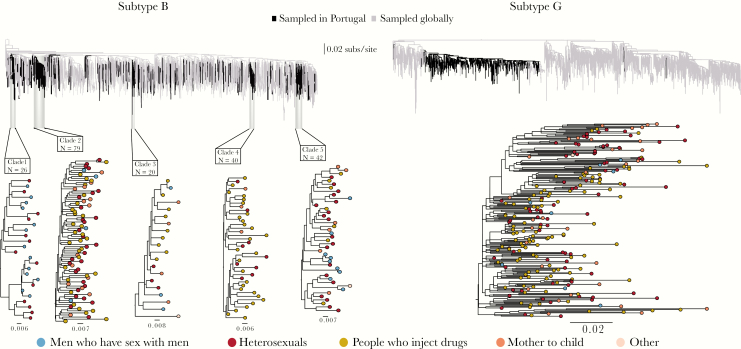
*Top*, Maximum likelihood (ML) phylogenetic trees reconstructed from the combined data sets, showing human immunodeficiency virus type subtype B (left) and subtype G (right). The black color indicates Portuguese sequences and is used to highlight their position on the global diversity trees. The highlighted clades are the clades of at least 10 sequences with a within-clade genetic distance <6% and a Shimodaira-Hasegawa–like statistical support >90%. *Bottom*, ML phylogenetic trees of the identified phylogenetic clades. The colors of the tips represent the transmission group of a patient from whom the sequence was sampled.

The maximum likelihood phylogenetic trees estimated for the subtype B and G combined data sets are shown in Figure 2. For subtype B, we identified 394 clades, of which 90 (representing 334 patients) included Portuguese sequences only. Five Portuguese circulating clades contained >10 sequences each and are denoted here as clades 1 (26 sequences), 2 (79 sequences), 3 (20 sequences), 4 (40 sequences), and 5 (42 sequences; Figure 2 and Table 1). Clades were located across the global diversity of subtype B, indicating that subtype B has been introduced multiple times to Portugal.

**Table 1.** Distribution of Sequences in Clades for Human Immunodeficiency Virus (HIV) Subtype B and Subtype G, by Transmission Risk Group

Risk Group	Subtype B Clade						Subtype G
	1	2	3	4	5	All Clusters	
MSM	13	4	3	…	9	29	8
PWID	…	32	9	28	7	76	110
HET	13	29	3	9	19	73	82
MTC	…	14	4	3	6	27	35
Other	…	…	1	…	1	2	1
Sequences, total no.	26	79	20	40	42	207	236
Sampling years	2001–2012	2001–2012	2001–2008	2001–2013	2001–2012	2001–2013	2001–2013

Abbreviations: HET, heterosexual; MSM, men who have sex with men; MTC, mother-to-child transmission; PWID, people who inject drugs.

### **Estimates of** R_e_**, Using Genetic Data**

**Figure 3.**
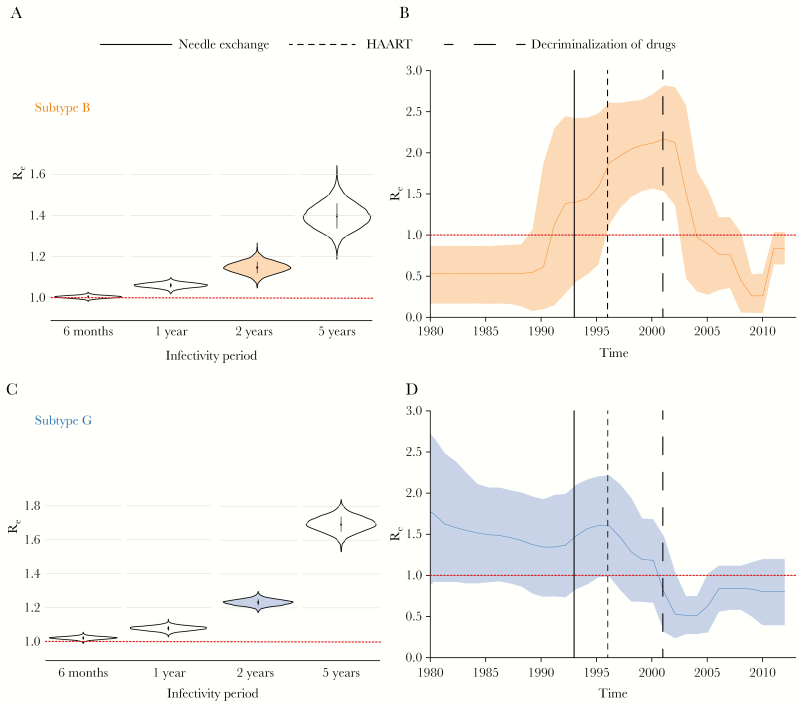
*A* and *C*, Effective reproductive number (R_e_) estimates obtained using the constant rate birth-death model (ie, model 1; constant R_e_) under different assumptions of infectious period (6 months–5 years; rate of becoming uninfectious, 2.0–0.2) for subtypes B (*A*) and G (*C*). The vertical lines in the middle of the violin plots represent the interquartile range. The filled with color violin plots (*A*) represent the value of R_e_ assuming the infectious period of 2 years. The horizontal red dotted line represents the epidemiological threshold (R_e_ = 1). *B* and *D*, R_e_ estimates obtained using the birth-death skyline model (ie, model 2) over 10 equidistant intervals between the tree height and the most recent tip in the analysis for subtype B (*B*) and G (*D*), assuming an infectious period of 2 years. The shaded area represents the 95% Bayesian credible interval. The horizontal red dotted line represents the epidemiological threshold (R_e_ = 1). Vertical lines correspond to the time of introduction of major interventions to prevent human immunodeficiency virus infection. HAART, highly active antiretroviral therapy.

To estimate temporal changes in R_e_, we used a suite of birth-death models that take into account shared ancestry and phylogenetic uncertainty. The constant-rate birth-death model (model 1) suggested that the number of secondary infections was on average marginally >1 over the course of the epidemic (Figure 3A and 3C). For example, assuming that an HIV-infected person remains infectious for a period of 2 years, after which this person has achieved virological suppression or has been removed from a population (via relocation or death), the median posterior estimate of R_e_ was 1.15 (95% Bayesian credible interval [BCI], 1.09–1.2) for subtype B and 1.23 (95% BCI, 1.2–1.27) for subtype G (Figure 3A and C). A sensitivity analysis specifying various infectious periods (from 6 months to 5 years; removal rate, 0.2–2.0) showed that R_e_ estimates decreased with shorter infectious periods but remained between 1 and 2 for all specified values (Figure 3A and 3C).

When using a BDSKY model (model 2), which allows changes in the rate of transmission through time, we found that the R_e_ of subtype B reached its peak in or around 2001 (R_e_, 2.17; 95% BCI, 1.53–2.82), after which, beginning in 2004, R_e_ decreased to <1 (95% BCI, 2002.5–2008; Figure 3B). For subtype G, the maximum value of R_e_ was observed in 1996 (R_e_, 1.6; 95% BCI, .98–2.2; Figure 3D). The mean R_e_ of subtype G decreased to <1 from 2000 onward (R_e_, 0.51; 95% BCI, .29–.75).

**Figure 4.**
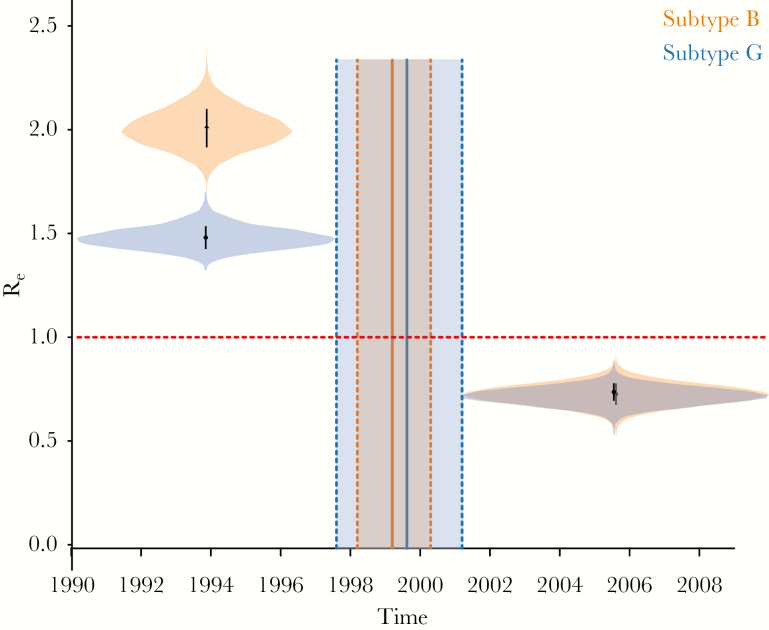
Effective reproductive number (R_e_) estimates obtained using the constant-shift-constant model (ie, model 3; 2 intervals) and the estimated time of change in the rate of epidemic spread for both subtypes. The violin plots of the R_e_ distribution before and after the time of the estimated change in viral spread rate are illustrated for both subtypes. The shaded area represents the 95% Bayesian credible interval. The horizontal red dotted line represents the epidemiological threshold (R_e_ = 1).

Finally, the 2-epoch birth-death model (model 3) revealed for both subtypes an initial phase of epidemic growth characterized by high R_e_ values (ie, well above 1), followed by a transition to a later phase of slower transmission. For subtype B, around 1999 (95% BCI, 1998–2000), R_e_ decreased from a median estimate of 1.92 (95% BCI, 1.75–2.1) to 0.62 (95% BCI, .52–.72; Figure 4). For subtype G, around 2000 (95% BCI, 1998–2001), R_e_ decreased from a median estimate of 1.49 (95% BCI 1.39–1.59) to 0.71 (95% BCI, .63–.8; Figure 4). Notably, model 3 indicates that the major transitions in the transmission dynamics of subtypes B and G occurred around the same time.

To investigate whether the same transmission dynamics patterns were observed in PWID populations, we repeated the subtype G analysis by including only sequences from PWID. This procedure resulted in R_e_ patterns for models 2 and 3 that were similar to those for the full subtype G data set (Supplementary Figure 1).

### Inferring Mixture of Transmission Behavior

To quantify the level of phylogenetic clustering by transmission behavior, we used 3 distinct approaches to measure the AI (Table 2) and 2 different discretization schemes, in which sequences were assigned to 5 or 3 different transmission risk groups. For the 5-group discretization scheme, BaTS indicated no significant transmission group structure in the subtype B clades, in contrast to the significant structure detected for subtype G (*P* < .01). The AI statistic suggests evidence of compartmentalization in subtype B clade 5 (AI, 0.77; bootstrap support, 0.97) and in the subtype G clade (AI, 0.84; bootstrap support, 0.98). Similar patterns for both subtypes were observed for the 3-transmission group discretization scheme.

**Table 2.** Association Indices (AIs) for Risk Group Compartmentalization Analysis

Subtype, Clade	All Risk Groups					3 Largest Risk Groups				
	Rescaled AI, BEAST	BaTS AI		Simmons AI		Rescaled AI, BEAST	BaTS AI		Simmons AI	
	Mean	Mean	*P*	Mean	Bootstrap	Mean	Mean	*P*	Mean	Bootstrap
Subtype B										
Clade 1	0.80	1.33	.21^a^	0.796^a^	0.67^a^	…	…	…	…	…
Clade 2	1.01	5.68	.09	0.95	0.53	0.99	5.28^a^	.06^a^	0.93^a^	0.65^a^
Clade 3	0.95	1.65	.28	0.93	0.51	0.97	1.51	.46	1.00	0.31
Clade 4	0.97	2.03	.22	0.957	0.39	…	…	…	…	…
Clade 5	0.80	3.01	.12	0.77^b^	0.97^b^	0.74	2.39^a^	.06^a^	0.72^b^	0.96^b^
Subtype G	0.95^b^	14.42^b^	<.01^b^	0.84^b^	0.98^b^	0.94^b^	13.86^b^	<.01^b^	0.84^b^	0.99^b^

^a^Statistically significant estimates at a *P* value < .1 or a bootstrap support > 0.6.

^b^Statistically significant estimates at a *P* value < .05 or a bootstrap support > 0.8.

**Figure 5.**
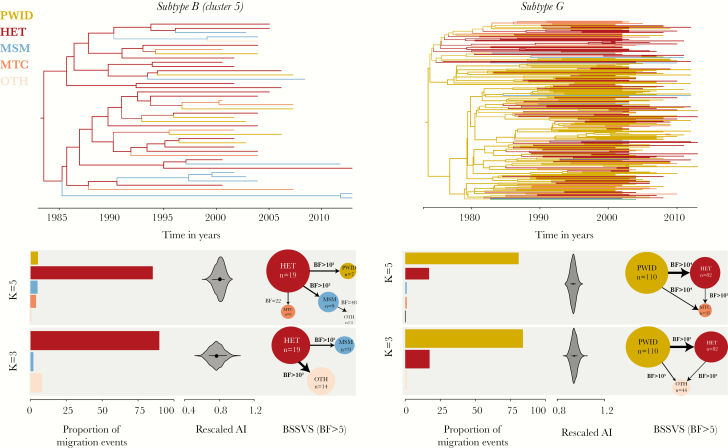
Results of the discrete trait analysis (DTA) for both human immunodeficiency virus subtypes. *Top*, Molecular clock phylogenetic trees reconstructed for subtype B clade 5 (left) and the subtype G clade (right). The color of the branches corresponds to the transmission group state; the width of the branches corresponds to the posterior probability support for the indicated transmission group state. *Bottom*, Results of the DTA for both subtypes, considering 5 and 3 transmission groups (k = 5 and k = 3, respectively). The colors represent the transmission group of a patient from whom the sequence was sampled. From left to right, *y*-axes show the proportion of migration events attributed to a transmission group, reconstructed using robust counts approach (numbers correspond to the number of sequences in the transmission group); the rescaled association index (AI); and Bayes factor (BF) values for the significant virus migration pathway. The size of the circles corresponds to the number of sequences in the analysis; the width of the arrows corresponds to the number of migration events for the corresponding pathways. HET, heterosexual; MSM, men who have sex with men; MTC, mother-to-child transmission; PWID, people who inject drugs.

We next performed DTA for subtype B clade 5 and for the large subtype G clade. The DTA results for subtype B clade 5 suggested that HET individuals disproportionally contributed to viral dissemination among transmission groups, as this group represented the inferred source of 82% of viral transmission group transition events (17 [95% BCI, 15–19] of 21 [95% BCI, 19–24]) between the transmission groups, while representing only 45% of the sequences (Figure 5 and Table 1). We next used a Bayesian stochastic search variable selection approach to identify the statistical support for virus movement among transmission risk groups [37]. We found that transmission group transitions with the highest support were from HET to MSM, from HET to PWID, from HET to MTC, and from MSM to OTH (BF, 976, 136, 48, and 22, respectively; Figure 5). When only 3 transmission groups were considered (HET, MSM, or Other), the estimated proportion of transmission group transition events from HET to Other was 90% (16 [95% BCI, 14–19] of 18 [95% BCI, 15–21]), and the pathways with the highest BF support were from HET to MSM and from HET to Other (BF, 1105 and 550, respectively).

Discrete diffusion analyses of subtype G suggest that PWID populations were the main contributors to viral transitions among transmission groups, accounting for 81% of transmission group transitions (82 [95% BCI, 60–96] of 101 [95% BCI, 90–110]), while representing 47% of the sequences (Figure 5 and Table 1). HET accounted for 17% of transmission group transitions (18; 95% BCI, 5–32) in the same data set, while composing 35% of the sequences. All other groups accounted for <1% of transmission group transition events. Pathways with the highest support were from PWID to HET, from PWID to MTC, and from HET to MTC, with BFs of 29 353, 29 353, and 116, respectively. Again, the same patterns were observed when only PWID, HET, and Other were considered.

## DISCUSSION

We analyzed HIV-1 genetic sequences from individuals in Portugal to better understand the dynamics of HIV-1 subtype B and G transmission in the country. Remarkably, our analyses of genetic data revealed that the rate of epidemic spread dropped significantly between 1998 and 2001. Moreover, we found little evidence for phylogenetic clustering by risk behavior, suggesting frequent mixing among transmission risk groups.

The estimated R_e_ of the Portuguese HIV-1 epidemic was on average above the epidemiological threshold of 1 throughout the history of subtype B and G epidemics in the country. When we estimated changes in R_e_ over time, we found that the growth rates of both subtypes were well above 1 for >10 years. R_e_ started to decline around 2001 for subtype B and around 1996 for subtype G. Importantly, the timing of this decline coincided with several major public health interventions around the mid-to-late 1990s, in particular the introduction of highly active ART in 1996. Interestingly, the estimated R_e_ of subtype G, which is dominated by transmissions in PWID, started to drop long before that of subtype B. This might be explained by a combined effect of highly active ART and harm-reduction programs introduced at the same time, as both syringe-exchange programs and OST have been shown to slow PWID-driven epidemics [39]. Furthermore, drug decriminalization in 2001 had a major effect on drug-related mortality in Portugal, with the number of cases decreasing from 80 to 16 between 2001 and 2012 [40]. Finally, the timing of a major decline in viral transmission dynamics for both subtypes, with a particularly rapid decrease in R_e_ for the PWID-driven subtype G, compared with subtype B, coincided with the decriminalization of drugs (Figure 3). This estimated decline in epidemic growth also coincided with a decline in the annual number of new HIV cases [5].

We used a novel hierarchical approach, implemented in a Bayesian framework. m This allowed us to combine information across 5 different smaller phylogenetic trees representing 5 subtype B clades and to estimate epidemiological parameters for this subtype without forcing all clades to belong to 1 phylogenetic tree. If all 5 clades were combined into 1 large phylogeny, then estimation of the deep branches in the tree (ie, the among-clade branches connecting the 5 clades) would add to statistical uncertainty, in part because of sequence saturation. Furthermore, the approach we used here allowed us to reliably estimate R_e_ even for the years prior to the sampling date of the oldest sequence in the analysis. This is an important advantage over methods that are based solely on the sequences’ sampling proportion, which might overestimate R_e_ for the year of the oldest samples by assuming that all individuals with infection diagnosed that year were infected the same year [15].

For subtype B specifically, it is important to remember that the findings of this study describe transmission dynamics of the main circulating subtype B clades but do not necessarily represent the Portuguese HIV-1 subtype B population as a whole. Even though the proportion of sequences from different transmission groups in our analysis is similar to that reported for the Portuguese epidemic before [5] (Supplementary Table 2), we cannot be sure that the transmission dynamic parameters inferred for the large clades would be identical for smaller clades.

We found phylogenetic evidence of frequent transmissions among different transmission groups, suggesting that interventions need to target epidemiologically linked groups simultaneously. Mathematical modeling suggests that halting new infections within “bridging” transmission groups may have the greatest impact in further reducing the incidence of HIV infection [41]. Historically, subtype B has been associated mostly with sexual HIV transmission in Portugal, while subtype G and CRF14_BG are more frequently reported in PWID [42]. Our results support the idea that PWID disproportionately contributed to the subtype G epidemic. After becoming established in PWID populations, subtype G spread to the more generalized HET population. For subtype B, no transmission group structure was observed for most of the clades, with only subtype B clade 5 showing a particularly high viral flow from HET to MSM. Notably, in our analysis, sequences from MSM grouped closely with sequences from HET and PWID, similar to the pattern previously reported from Minho Province in northern Portugal [7]. In other European countries and the United States, however, sequences sampled from MSM tend to form isolated clades that are poorly mixed with other transmission groups [43–47]. However, because we relied on self-reported transmission risk group information, it is possible that the virus lineage movement between HET and MSM results, at least in part, from the misclassification of HET infections and the underreporting of MSM contacts, as previously shown in the United Kingdom and Nordic countries [48, 49].

Portugal has a well-developed surveillance system that enables timely identification of new HIV infections, large-scale ART provision, and routine antiretroviral drug resistance testing, which forms the basis of a large national database of sequences from the genomic regions encoding protease and reverse transcriptase. Unfortunately, the majority of the sequences from the HIV Portuguese database are unlinked to additional epidemiological information, such as transmission risk group and stage of infection. Analysis of between- and within-risk group transmission is critical to the design of optimal strategies that can directly impact the progress of an HIV epidemic in a country [44]. Improving the linkage between patient records and sequence data would allow larger-scale studies to be undertaken that could directly influence public health strategies aimed at reducing HIV transmission in real time, similar to what has recently been done in Canada [50].

In conclusion, our study shows how genetic data can help us to address challenging epidemiological questions and serve as evidence for public health decision-making. Further improvements both in statistical approaches and in genomic surveillance in healthcare settings can facilitate and encourage the application of phylogenetics to eliminate new infections among distinct transmission groups.

## Supplementary Data

Supplementary materials are available at *The Journal of Infectious Diseases* online. Consisting of data provided by the authors to benefit the reader, the posted materials are not copyedited and are the sole responsibility of the authors, so questions or comments should be addressed to the corresponding author.

jiz085_suppl_Supplementary_Figure_1

jiz085_suppl_Supplementary_Figure_2

jiz085_suppl_Supplementary_Figure_Legends

jiz085_suppl_Supplementary_Tables

jiz085_suppl_Supplementary_Text

## Notes

***Acknowledgments.* ** We thank all patients from whom data were collected and analyzed; the European Society for Translational Antiretroviral Research, for providing us with genetic sequence data for this study; Anne Cori of Imperial College London and Christophe Fraser of the University of Oxford, for early discussions.

***Financial support*. **This work was supported by the New College of the University of Oxford (Juliana Cuyler Matthews Junior Research Fellowship to T. I. V.); the Royal Society and Wellcome Trust (Sir Henry Dale Fellowship [grant 204311/Z/16/Z] to N. R. F.); the European Research Council, under the European Commission Seventh Framework Programme (FP7/2007–2013)/European Research Council (grant agreement 614725-PATHPHYLODYN; to O.G. P and L. d. P.); the Max Planck Society (to D. K.); European Funds (grant ‘Bio-Molecular and Epidemiological Surveillance of HIV Transmitted Drug Resistance, Hepatitis Co-Infections and Ongoing Transmission Patterns in Europe—BEST HOPE—[project funded through HIVERA: Harmonizing Integrating Vitalizing European Research on HIV/AIDS; grant 249697]); L’Oréal Portugal Medals of Honor for Women in Science 2012 (financed through L’Oréal Portugal, Comissão Nacional da Unesco and Fundação para a Ciência e Tecnologia [ FCT]); by FCT for funds to GHTM-UID/Multi/04413/2013; by the MigrantHIV project (financed by FCT; PTDC/DTP-EPI/7066/2014); by Gilead Génese HIVLatePresenters; the Research Foundation Flanders (FWO G.0692.14); and the VIROGENESIS project, which receives funding from the European Union’s Horizon 2020 Research and Innovation Programme (grant agreement 634650).

***Potential conflicts of interest.* **All authors: No reported conflicts of interest. All authors have submitted the ICMJE Form for Disclosure of Potential Conflicts of Interest. Conflicts that the editors consider relevant to the content of the manuscript have been disclosed.

Presented in part: 24th International HIV Dynamics and Evolution Conference, Isle of Skye, United Kingdom; 23–26 May 2017; 2nd Virus Genomics and Evolution Conference, Cambridge, United Kingdom, 18–20 June 2018.
