# Supplementary figures and images for: Tracing the Impact of Public Health Interventions on HIV-1 Transmission in Portugal Using Molecular Epidemiology
Source: J Infect Dis. 2019 Feb 26;220(2):233–43. doi: 10.1093/infdis/jiz085 (PMC6581889; doi:10.1093/infdis/jiz085)

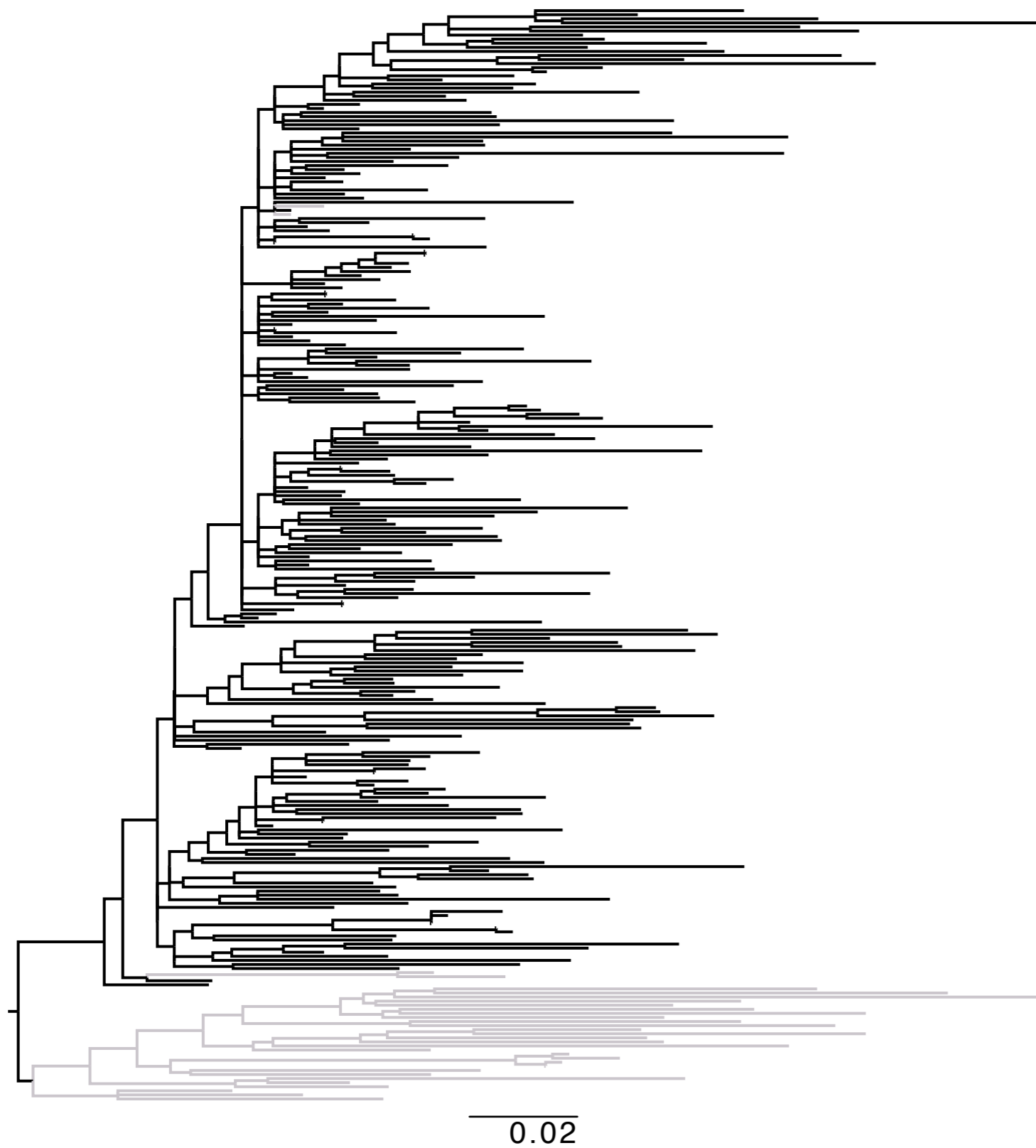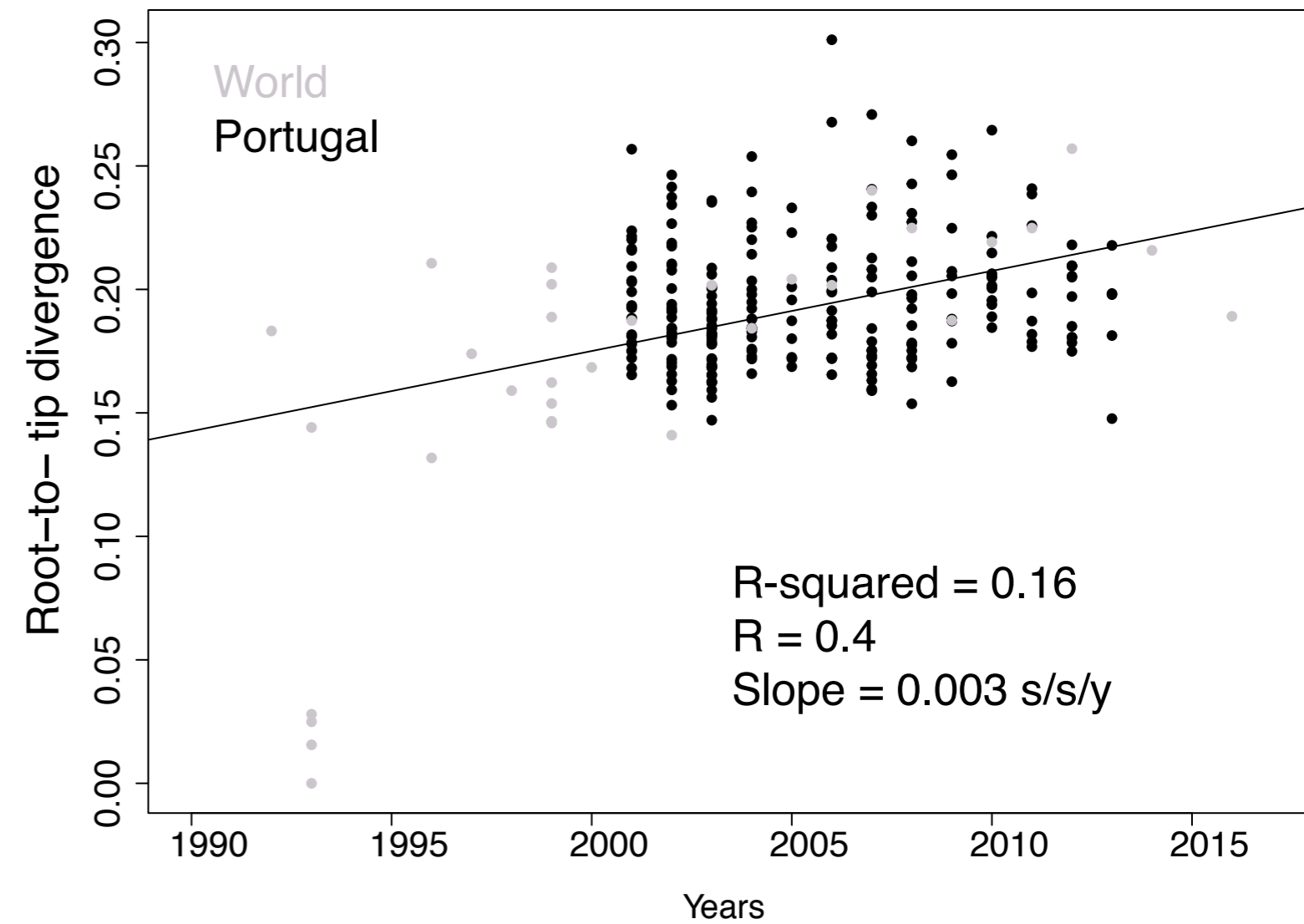

Supplement: jiz085_suppl_Supplementary_Figure_1 [file jiz085_suppl_supplementary_figure_1.pdf]

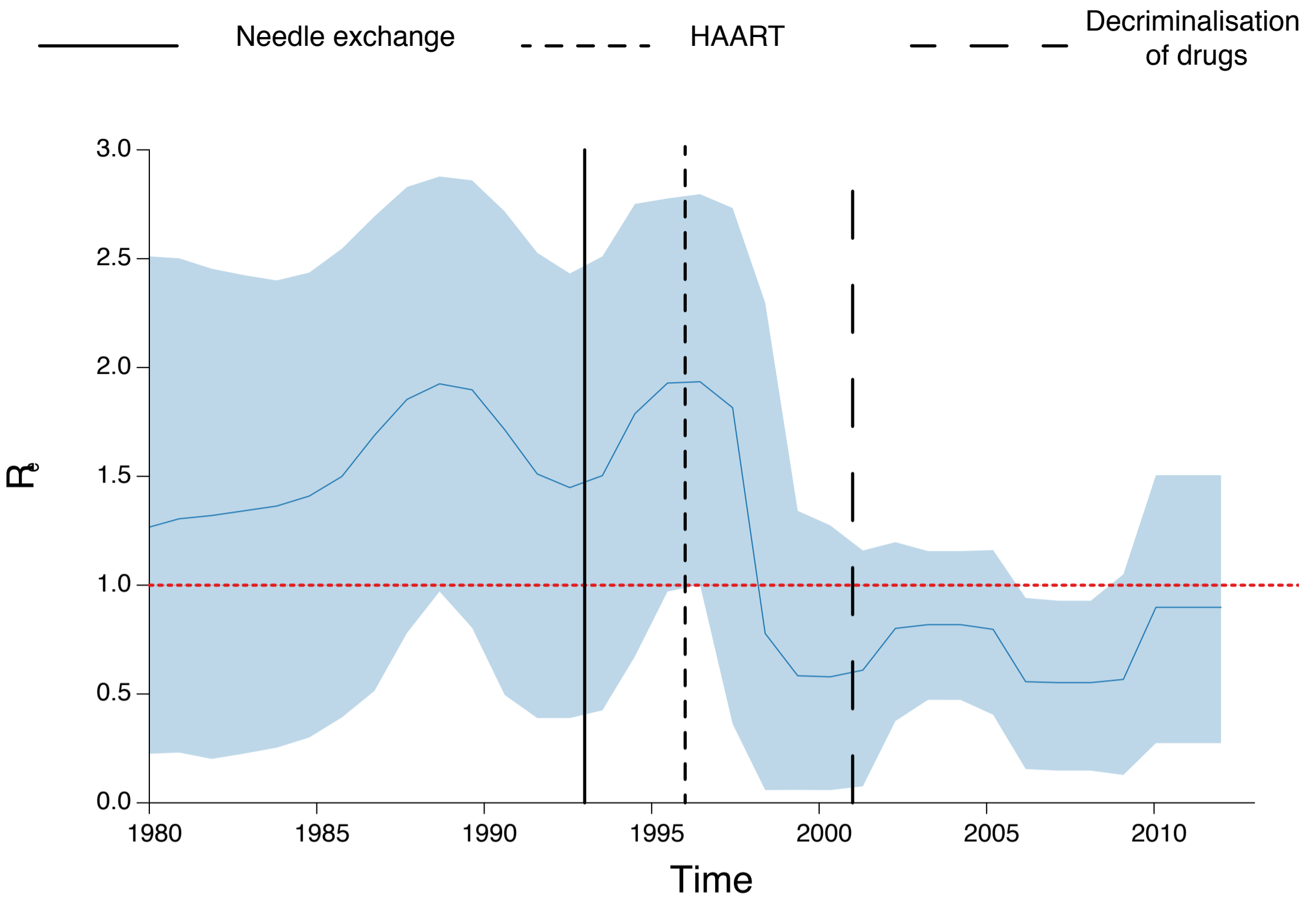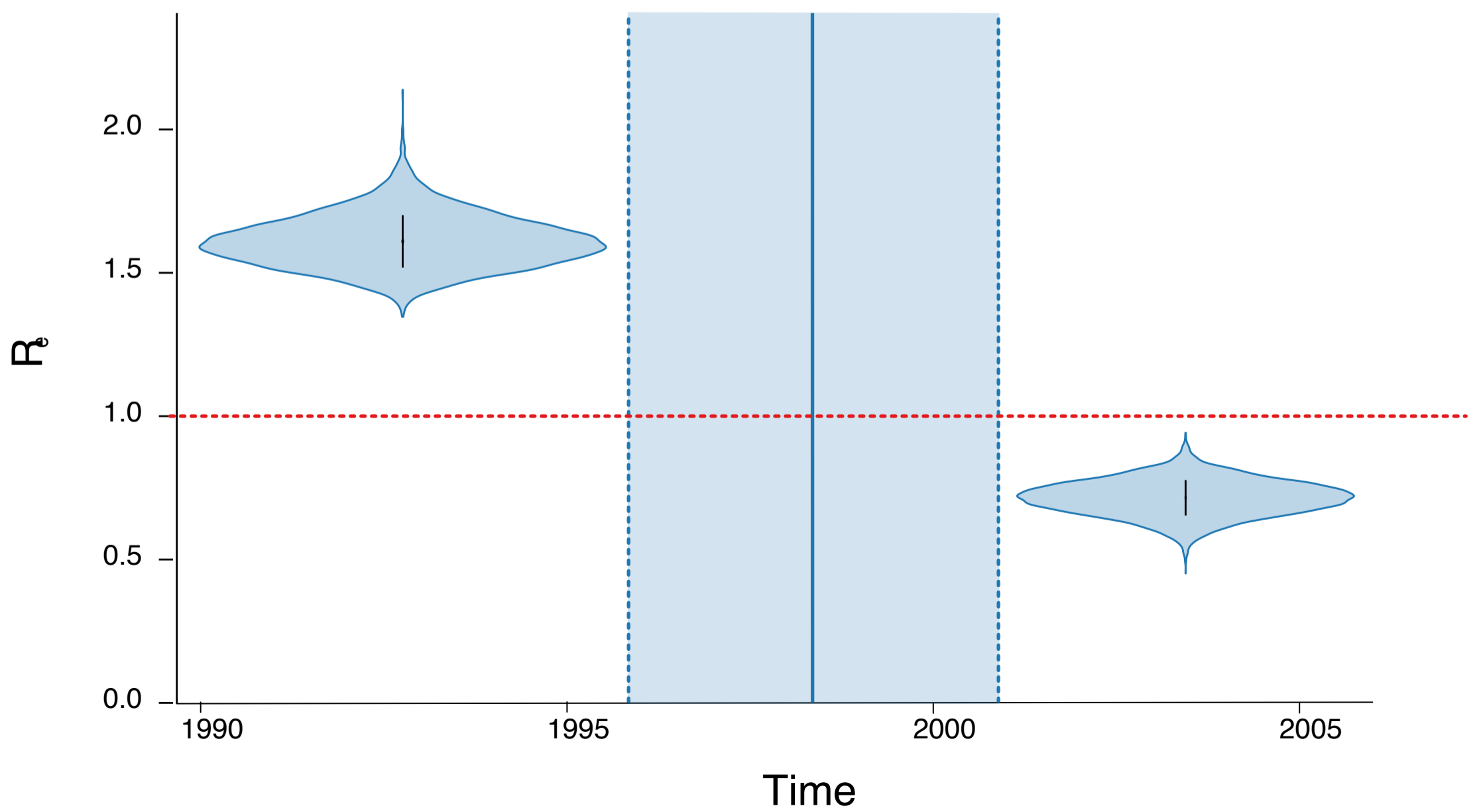

Supplement: jiz085_suppl_Supplementary_Figure_2 [file jiz085_suppl_supplementary_figure_2.pdf]
