# Supplementary material for: Tracing the Impact of Public Health Interventions on HIV-1 Transmission in Portugal Using Molecular Epidemiology
Source: J Infect Dis. 2019 Feb 26;220(2):233–43. doi: 10.1093/infdis/jiz085 (PMC6581889; doi:10.1093/infdis/jiz085)
Supplement: jiz085_suppl_Supplementary_Figure_Legends [file jiz085_suppl_supplementary_figure_legends.docx]

**Suppl. Fig 1.** Subtype G R_e_ estimated using BD Models 2 (top) and 3 (bottom) using only PWID sequences that could be traced to the root of the phylogenetic tree and had a high posterior probability (>0.85%) for all internal nodes (N=99). The shaded area represents Bayesian credible interval. The horizontal red dotted line represents the epidemiological threshold (R_e_=1). Top: Vertical lines correspond to the time of introduction of major HIV preventive interventions. Bottom: The violin plots of R_e_ distribution before and after the time of estimated change in the rate of epidemic spread. The vertical lines in the middle of the violin plots represent the interquartile range.

**Suppl. Fig 2.** Maximum Likelihood phylogenetic tree of the Portuguese subtype G dataset (N=236) and all publicly available from the Los Alamos database subtype G sequences sampled prior to 2001 (N=32) (left). Results of the Tempest analysis for the tree on the right. R – correlation coefficient.
