# Supplementary material for: Tracing the Impact of Public Health Interventions on HIV-1 Transmission in Portugal Using Molecular Epidemiology
Source: J Infect Dis. 2019 Feb 26;220(2):233–43. doi: 10.1093/infdis/jiz085 (PMC6581889; doi:10.1093/infdis/jiz085)
Supplement: jiz085_suppl_Supplementary_Text [file jiz085_suppl_supplementary_text.docx]

**Supplementary Text to “Tracing the Impact of Public Health Interventions on HIV-1 Transmission in Portugal Using Molecular Epidemiology”**

**Data and alignment**

The Portuguese HIV database includes *pol* nucleotide sequences with the following associated information: hospital of attendance, date of sample collection, and self-reported transmission group (for some sequences). Only sequences with available transmission group information were used in this analysis. Such data were retrieved from patients who attended 18 hospitals located over the Portuguese mainland and the Madeira Archipelago. If two sequences were available for the same patient, only the sequence with the earlier sampling date was used. Since subtype G and CRF14_BG do not have breakpoints in *pol* nucleotide sequences, a stringent subtyping approach was used. All sequences were subtyped using Rega subtyping tool version 3 ^[1]^ and COMET ^[2]^ and only sequences with concordant subtype assignments were considered for subsequent analyses. Results from the manual phylogenetic subtyping analyses previously performed ^[1]^ were also considered. Potential CRF14_BG sequences or BG recombinants were excluded from the analyses.

We aligned all sequences (including the reference) using Clustal Omega software^[3]^. Initial alignments were 879 and 1020 nucleotides (nt) long for subtypes B and G, respectively. We then manually edited the alignment using MEGA7 program^[4]^ to delete codon positions associated with drug resistance (RT: 41, 65, 67, 69, 70, 74, 75, 77, 100, 101, 103, 106, 115, 116, 151, 179, 181, 184, 188, 190, 210, 215, 219, 225, 230; PR: 23, 24, 30, 32, 46, 47, 48, 50, 53, 54, 73, 76, 82, 83, 84, 85, 88, 90) ^[5]^. The final alignments were 804 and 945 nt long for subtypes B and G, respectively.

**Clades definition**

All the same clades, except the largest (clade 2), were also identified when a more conservative clade definition was used (genetic distance threshold <4.5%). Parts of the clade 2 were found at the 4.5% distance threshold, but these were comprising less than 10 sequences and, thus, did not qualify for our “circulating clade” definition.

**TempEst analysis**

To estimate temporal structure we regressed genetic divergence against sampling dates ^[6]^. We find that each subtype B clade contained a moderate temporal signal as indicated by correlation coefficients, R, of 0.54, 0.22, 0.37, 0.35, and 0.33 for clades 1 to 5, respectively (Suppl. Table 3).

For the subtype G, the molecular clock signal was weak (Suppl Table 3). Thus, we used a normal prior on the time of the most recent common ancestor (TMRCA). To increase temporal resolution of our dataset, we downloaded all publicly available subtype G sequences from LANLdb collected before 2001 (N=32) and added them to the 236 Portuguese sequences (full dataset, N=268). We reconstructed the Maximum Likelihood (ML) tree for the full dataset using RAxML (Suppl Fig. 1). This allowed us to improve the root-to-tip correlation (R), estimated using Tempest program, from R=0.23 to R=0.40. We then used BEASTv1.8.4 to estimate the TMRCA from the full dataset under a strict molecular clock model, a Hasegawa-Kishino-Yano (HKY) nucleotide substitution model and a Skygrid coalescent tree prior^[7]^. The obtained TMRCA estimate for the subtype G Portuguese clade was 1977 (standard deviation of 2.1); this was used as a normal prior on subsequent subtype G analyses.

**BDM analyses**

We used strict molecular clock model and the HKY+G nucleotide substitution model ^[8]^. Since the times of the most recent sampling dates and the TMRCAs will differ between clades, we used clade 3 to define the shift-times (times of the change of BDSKY intervals) of the birth-death skyline model because the time of this clade’s most recent sample (2008) is older than the time of the most recent sample in the other clades. Thus, the time of the shifts was calculated on a scale between the TMRCA and the youngest tip in clade 3. We used TreeSlicer package in BEAST2 for this analysis (https://github.com/laduplessis/skylinetools).

The following assumptions were encoded as priors in the Bayesian analyses: (i) lognormal prior with mean 1.0 and standard deviation 1.25 was placed on the clock rate; (ii) lognormal prior with mean 1.0 and standard deviation 1.25 was used for the kappa parameter in HKY model; (iii) uniform prior between the TMRCA of the sampled tree and 100 years before the most recent sample was used for the origin of each clade, (iv) lognormal distribution with mean 1.0 and standard deviation 1.25 was placed on R_e_, (v) sampling proportion was set to 0 between the TMRCA and the oldest sample, which was in 2001 for all clades and both subtypes; after 2001 (between 2001 and the most recent sample in each clade) a Beta distribution with parameters α=1.0 and β=10.0 was used for the sampling proportion, (vi) for the constant-shift-constant model a uniform prior between 1992 and 2007 was placed on the shift date; (viii) for subtype B we placed a uniform distribution truncated between the 95% Bayesian credible intervals as previously estimated in Abecasis et al.^[9]^ (viii) for subtype G we used a normally distributed TMRCA prior with mean 1976.9 and standard deviation 2.1 as described above. Finally, the removal rate for all the BD models was fixed to 0.5 years^-1^ (corresponding to an average infectious period of 2 years). In sensitivity analyses we varied the removal rate between 0.1 and 4.0 years^-1^, resulting in average infectious periods between 3 months and 10 years.

Additionally, for all the BDSKY analyses we chose 0.05 as a starting value for the sampling proportion parameter, and allowed this parameter to vary between 0 and 0.2. This value was chosen based on the number of subtype B and subtype G sequences in each clade per year relative to the number of registered HIV-1 cases that year and the proportion of subtype B (≈40%) and subtype G (≈20%) cases ^[10]^. The proportion of sequenced sequences never exceeded 9% by our estimates (Suppl. Table 4); thus, 0-0.2 was a reasonable interval for this parameter.

To increase computational speed, we ran BEASTv.1.8.4 analyses using the BEAGLEv3.1 library ^[11]^.

*Subtype B analysis for combined dataset*

We performed BDSKY analyses (Model 2) as described in the main text with all five HIV-1 subtype B clades analysed as a single phylogenetic tree to test the robustness of our hierarchical model approach. The same evolutionary models and prior parameters were kept for this analysis. Using this single-tree approach, the median posterior R_e_ for subtype B was estimated to be 1.2 (95% Bayesian credible interval, BCI, 1.17 – 1.24). The BDSKY model showed that the R_e_ started declining after 1998 (95% BCI 1999 – 2000), and dropped significantly below 1 after 2005. Further, we estimated the time of the major shift in transmission dynamics to be in 1999 (95% BCI 1998 – 2000), with Re being 2.00 (95% BCI 1.80 – 2.20) before and 0.57 (95% BCI 0.47-0.66) after the shift. All these estimates are similar to those obtained from the hierarchical model, supporting the robustness of our findings.

*Subtype G analysis with PWID sequences only*

For subtype G, we ran additional analysis including PWID sequences only using Models 2 and 3. Specifically, we chose sequences that could be traced to the root of the phylogenetic tree and had a high posterior probability (>0.85%) for all internal nodes (N=99) as previously described ^[12]^. The reconstructed BDSKY from the PWID-only sequences showed similar patterns as the BDSKY reconstructed from the full dataset, with a rapid decline after 1996 (Suppl. Fig.2). In Model 3 R_e_ transitioned from ~1.61 (95% BCI 1.44 to 1.79) to 0.71 (95% BCI 0.59 to 0.83), which is very similar to the full dataset values (Fig.4), but with a broader confidence interval, most likely because of the small number of sequences. The time of the major shift in transmission dynamics was estimated to be in 1998 (95% BCI 1996-2001), which is also similar to the estimates obtained by the full dataset.

**Discrete trait analysis**

*Association index analyses*

Since the AI value typically depends on the number of tips in a phylogeny, we used several methods to estimate this parameter. First, we used BEAST to obtain AI normalised by the values obtained by tip randomisation (0 to 1, with 0=maximum structure and 1=panmixia), which allowed direct comparison of AI between clades of different sizes; ii) we used BaTS ^[13]^ to obtain statistical support (p-values) for how the mean posterior estimate of AI from the empirical tree distribution is different from the AI obtained by randomly re-assigning traits at the tips 100 times; iii) we used HyPhy ^[14]^ to estimate Simmonds’ AI, which assesses compartmentalisation by weighting internal nodes based on their position on the tree, giving less value to the nodes near the root; it also allowed us to obtain statistical support (bootstrap values) for the observed AI ^[15]^. We chose topology-based methods that incorporate phylogenetic uncertainty (like BaTS and Simmons AI) since these methods tend to perform best when studying shorter HIV-1 sequences like the ones used in our analysis ^[16]^.

*BEAST discrete trait analyses*

We used the following priors for this analysis: (i) an asymmetric continuous-time Markov chain prior was used for the transmission group clock rate^[17]^; (ii) a uniform prior was used for the transmission group frequencies; (iii) a gamma prior with shape parameter 1.0 was used for the transmission group rates; (iv) a uniform prior was used for the root frequencies.

*Empirical tree distribution*

We resampled posterior trees obtained from the BDSKY analyses, resulting in a distribution of 2,000 trees for each clade in the analysis (burn-in 10% and 20% for subtypes B and G, respectively)^[18]^.

**References:**

1. Pineda-Pena AC, Faria NR, Imbrechts S, Libin P, Abecasis AB, Deforche K, et al. Automated subtyping of HIV-1 genetic sequences for clinical and surveillance purposes: performance evaluation of the new REGA version 3 and seven other tools. *Infect Genet Evol* 2013; 19:337-348.

2. Struck D, Lawyer G, Ternes AM, Schmit JC, Bercoff DP. COMET: adaptive context-based modeling for ultrafast HIV-1 subtype identification. *Nucleic Acids Res* 2014; 42(18):e144.

3. Sievers F, Wilm A, Dineen D, Gibson TJ, Karplus K, Li W, et al. Fast, scalable generation of high-quality protein multiple sequence alignments using Clustal Omega. *Mol Syst Biol* 2011; 7:539.

4. Kumar S, Stecher G, Tamura K. MEGA7: Molecular Evolutionary Genetics Analysis Version 7.0 for Bigger Datasets. *Mol Biol Evol* 2016; 33(7):1870-1874.

5. Bennett DE, Camacho RJ, Otelea D, Kuritzkes DR, Fleury H, Kiuchi M, et al. Drug Resistance Mutations for Surveillance of Transmitted HIV-1 Drug-Resistance: 2009 Update. *Plos One* 2009; 4(3).

6. Rambaut A, Lam TT, Max Carvalho L, Pybus OG. Exploring the temporal structure of heterochronous sequences using TempEst (formerly Path-O-Gen). *Virus Evol* 2016; 2(1):vew007.

7. Gill MS, Lemey P, Faria NR, Rambaut A, Shapiro B, Suchard MA. Improving Bayesian population dynamics inference: a coalescent-based model for multiple loci. *Mol Biol Evol* 2013; 30(3):713-724.

8. Hasegawa M, Kishino H, Yano T. Dating of the human-ape splitting by a molecular clock of mitochondrial DNA. *J Mol Evol* 1985; 22(2):160-174.

9. Abecasis AB, Vandamme AM, Lemey P. Quantifying differences in the tempo of human immunodeficiency virus type 1 subtype evolution. *J Virol* 2009; 83(24):12917-12924.

10. INSA DdDaId. Infeção VIH SIDA: a situação em Portugal a 31 de dezembro de 2014. In. Lisboa: Instituto Nacional de Saúde Doutor Ricardo Jorge; 2015.

11. Ayres DL, Darling A, Zwickl DJ, Beerli P, Holder MT, Lewis PO, et al. BEAGLE: An Application Programming Interface and High-Performance Computing Library for Statistical Phylogenetics. *Systematic Biology* 2012; 61(1):170-173.

12. Faria NR, Rambaut A, Suchard MA, Baele G, Bedford T, Ward MJ, et al. HIV epidemiology. The early spread and epidemic ignition of HIV-1 in human populations. *Science* 2014; 346(6205):56-61.

13. Parker J, Rambaut A, Pybus OG. Correlating viral phenotypes with phylogeny: accounting for phylogenetic uncertainty. *Infect Genet Evol* 2008; 8(3):239-246.

14. Pond SLK, Frost SDW, Muse SV. HyPhy: hypothesis testing using phylogenies. *Bioinformatics* 2005; 21(5):676-679.

15. Wang TH, Donaldson YK, Brettle RP, Bell JE, Simmonds P. Identification of shared populations of human immunodeficiency virus type 1 infecting microglia and tissue macrophages outside the central nervous system. *J Virol* 2001; 75(23):11686-11699.

16. Zarate S, Pond SL, Shapshak P, Frost SD. Comparative study of methods for detecting sequence compartmentalization in human immunodeficiency virus type 1. *J Virol* 2007; 81(12):6643-6651.

17. Edwards CJ, Suchard MA, Lemey P, Welch JJ, Barnes I, Fulton TL, et al. Ancient hybridization and an Irish origin for the modern polar bear matriline. *Curr Biol* 2011; 21(15):1251-1258.

18. Faria NR, Rambaut A, Suchard MA, Baele G, Bedford T, Ward MJ, et al. The early spread and epidemic ignition of HIV-1 in human populations. *Science* 2014; 346(6205):56-61.
